# Preparing Unbiased T-Cell Receptor and Antibody cDNA Libraries for the Deep Next Generation Sequencing Profiling

**DOI:** 10.3389/fimmu.2013.00456

**Published:** 2013-12-23

**Authors:** Ilgar Z. Mamedov, Olga V. Britanova, Ivan V. Zvyagin, Maria A. Turchaninova, Dmitriy A. Bolotin, Ekaterina V. Putintseva, Yuriy B. Lebedev, Dmitriy M. Chudakov

**Affiliations:** ^1^Shemyakin-Ovchinnikov Institute of Bioorganic Chemistry, Russian Academy of Science, Moscow, Russia; ^2^CEITEC, Masaryk University, Brno, Czech Republic

**Keywords:** TCR repertoires, BCR repertoires, NGS applications, cDNA libraries, MiTCR, IG repertoires, T-cell receptor, T-cell receptor repertoire

## Abstract

High-throughput sequencing has the power to reveal the nature of adaptive immunity as represented by the full complexity of T-cell receptor (TCR) and antibody (IG) repertoires, but is at present severely compromised by the quantitative bias, bottlenecks, and accumulated errors that inevitably occur in the course of library preparation and sequencing. Here we report an optimized protocol for the unbiased preparation of TCR and IG cDNA libraries for high-throughput sequencing, starting from thousands or millions of live cells in an investigated sample. Critical points to control are revealed, along with tips that allow researchers to minimize quantitative bias, accumulated errors, and cross-sample contamination at each stage, and to enhance the subsequent bioinformatic analysis. The protocol is simple, reliable, and can be performed in 1–2 days.

## Introduction

Next generation sequencing (NGS) technologies opened a breathtaking opportunity to perform deep analysis and comparative studies of the T-cell receptor (TCR) and antibody (IG) repertoires of the human donors and model animals, as well as of the various sorted, separated, or cultured lymphocyte subsets of interest (1–13). Still, rational NGS-analysis of such immune repertoires is critically dependent on the library preparation protocols, starting from a lymphocytes/PBMC sample and ending with the amplification of individual TCR/IG segment encoding molecules on the solid phase of a sequencing machine. Multiple sampling bottlenecks, PCR biases, and cross-contamination at different stages lie in wait to trick a researcher on his way to get the deep, clear, and congruent data.

While studying autoimmunity and hematopoietic stem cell transplantation therapy (10, 14–17), we have optimized cDNA-based protocol that allows unbiased pre-sequencing amplification of the human and murine, alpha- and beta-TCR, as well as IG heavy chain gene libraries. The protocol employs a specific oligonucleotide to prime cDNA synthesis, and template switching effect to form a universal 5′-adapter and to introduce sample barcode at the very first stage of library preparation. Subsequent two-step PCR amplification is performed with universal pairs of primers for the whole library using step-out plus PCR-suppression effect (18) on the 5′-end and nested PCR (19) on the 3′-end of the library (16).

This approach allows efficient and unbiased amplification of millions of the TCR/IG mRNA molecules in only 27–30 (21–24 considering dilution factor, see below) PCR cycles, thus providing sufficient starting material for the deep NGS-analysis of complex lymphocyte samples. Current protocol is optimal for the sequencing on Illumina MiSeq/HiSeq platforms and Roche 454 platforms.

Here we report the upgraded and tested protocol in a ready-to-use format with the technical details required for the method to be easily and uniformly reproduced in any laboratory.

## Advantages of cDNA Libraries and 5′-Template Switch

Starting with cDNA synthesis using 5′-template switching (16, 20, 21) has at least two decisive advantages in comparison with the genomic DNA-based approaches (2, 12).

**Figure 1:**
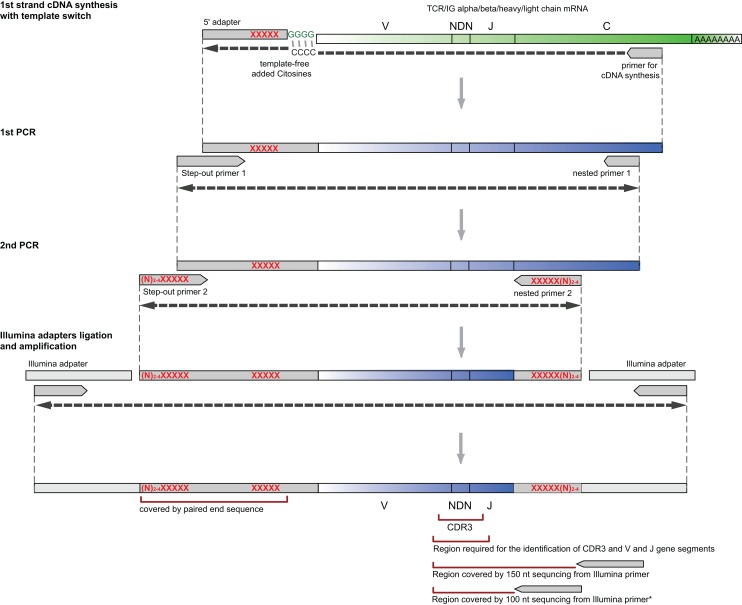
**Flow-chart of the library preparation protocol from RNA and to NGS-ready PCR product**. XXXXX: optional sample barcodes (see Sample Barcoding in Appendix for details and Supplementary Material for barcodes). *For TCR alpha/beta profiling with 100 nt sequencing length, multiplexed J-segment-specific primers should be used as a reverse primer in the second PCR amplification step as described in section “Next Generation Sequencing Options.”

First, the whole diversity of variable chains (up to approximately 100 different V gene segment variants^1^, can be amplified using just a pair (for TCRs) or a simple multiplex set (for IGs) of oligonucleotides, specific to the template switch adapter on the 5′-end and to the constant gene segments on the 3′-end of the library (Figure 1).

In contrast, the approaches starting with the genomic DNA require multiplex primer sets to be used both at the 5′ V gene segments’ end, and at the 3′ introns/J-segments end of the library (2). Moreover, a subsequent nested PCR amplification, which requires another set of multiplex primers, can be necessary to obtain pure TCR or IG library from genomic DNA. Multiplexing inevitably leads to dramatic bias in relative efficiency of amplification of different variable segments and thus to the loss of quantitative information, and complete loss of some of the rare clonotypes (10, 16, 22, 23).

Second, abundant copies of mRNAs encoding TCR or IG chains comprise an essential portion of the total lymphocyte RNA. This practically results in an efficient amplification of a deep library starting from 10^6^ mRNA molecules from a 3 μg of total RNA sample purified from three million PBMC cells (10). cDNA synthesis reaction can be performed in a volume of 10–15 μl in a single PCR tube (see Protocol), allowing multiple parallel experiments to be carried out.

In contrast, amplification of the TCR/IG library starting from 15 μg of genomic DNA of the same three million PBMC sample requires PCR to be carried out in larger volumes (since no more than 0.5 μg of genomic DNA can be taken for a 50 μl PCR reaction), and still does not provide comparable PCR efficiency, i.e., essential portion of the original sample diversity is lost due to the stochastic character of PCR, inevitably missing rare molecules.

## Limitations of the Use of cDNA Libraries and 5′-Template Switch

We have recently demonstrated that cDNA-based template switching protocol is highly quantitative at the ensemble level – the level of relative TRBV gene segments’ frequencies (10). Indeed, PCR bias is minimized and the whole approach is quite quantitative in respect of relative abundance of mRNA molecules at start and sequencing reads at the end of analysis pipeline. However, it should be noted that individual T-cell or B-cell clones can potentially be characterized by higher or lower expression levels of TCR or IG mRNA (24, 25). This limitation should be kept in mind when using NGS data for the estimation of particular lymphocyte clones’ relative abundance.

It is generally important that the cells being analyzed “feel fine” and contain a sufficient amount of TCR/IG mRNA. Therefore, it is preferable to purify total RNA from a freshly isolated cell sample for the native analysis. For the frozen samples, overnight incubation of thawed cells in presence of IL2 (Roche, 15 U/ml) leads to at least twofold increase of TCR genes RNA expression levels (our unpublished observations).

Differences in the efficiency of reverse transcription and template switching may lead to a different number of cDNA molecules read per T- or B-cell. Therefore, it is important to use the same reverse transcriptase and 5′-template switch adapter and carry out all the procedures in identical experimental conditions to obtain results that can be further accurately compared at the deep level (e.g., in an analysis of relative diversity of naïve T cells or a PBMC sample, etc.).

## Experimental Design: Cells, Numbers, and Bottlenecks

The desirable depth of TCR or IG repertoire analysis depends on the particular experimental questions raised. For example, application of the current protocol for the deep analysis of a PBMC sample containing 10^6^ T cells will provide quantitative data on those TCR clonotypes that constitute at least 0.01–0.1% of all T cells in a sample (100–1000 T cells) (10). The majority (>95%) of TCR clonotypes constituting at least 0.001% (at least 10 T cells) will be sequenced, while approximately 20–40% of TCR clonotypes represented by a single T cell in a sample may be lost (estimated according to our quantitative experiments, depends on the reverse transcriptase used). Preferably, all the synthesized cDNA should be used for the first PCR amplification step. Second PCR should result in sufficient amount of target PCR product in a reasonable number of amplification cycles (see Protocol). The desirable number of output CDR3-containing high quality sequencing reads is at least 2 × 10^6^ per sample (see Protocol and Expected Results).

Much smaller bottleneck limits should be quite sufficient for the majority of the experimental tasks concerning more specific subpopulations of lymphocytes characterized by lower diversity [such as sorted antigen-specific T cells (26) or B cells (27)]. For example, 10,000 lymphocytes, 10 ng high quality total RNA, no more than 21 first PCR cycles, no more than 20 s PCR cycles (see Protocol and Expected Results), and at least 30,000 CDR3-containing sequencing reads (ideally 100,000 reads to achieve over-sequencing) per sample may be sufficient to identify most TCR/IG clonotypes in a low-complexity sample. It is preferable to use reverse transcriptase with high 5′-template switching efficiency (e.g., SMARTScribe, Clontech) when small cell samples/RNA amounts are analyzed.

## Experimental Design: Sample Barcodes, Multiplexed Sequencing, Cross-Contamination

Since as few as 30,000 sequencing reads per sample may be sufficient for many experimental tasks in immune repertoire’s profiling, and, for example, paired end 150 bp Illumina MiSeq run can produce more than five million good quality TCR/IG CDR3 reads, a researcher may be often interested in sequencing multiple samples in a single run. At the same time, ligating Illumina sample barcodes to 10 or more samples is rather expensive and laborious. Our design suggests that sample barcodes can be introduced within the 5′-template switch adapter during cDNA synthesis and/or second PCR amplification steps (see Figure 1). Samples with the barcodes inside can be then combined in equal (or unequal, if it is desirable to get more reads for some samples) proportions, and Illumina adapters can be ligated to the resulting pooled PCR library of approximately 500–600 bp length (see Protocol and Sample Barcoding in Appendix).

Sample barcodes on both ends of the library allow to eliminate most cross-contaminations between the samples sequenced in the same run/lane that may occur during the amplification of the combined sample after adapters’ ligation, and potentially in course of bridge amplification on the solid phase of the sequencing machine.

To avoid contamination on the earlier stages of pre-sequencing library preparation, all procedures, including: RNA purification, cDNA synthesis, first and second PCR preparation – should be performed in separate clean PCR boxes.

## Protocol

### Preparing starting material – total RNA

1. Use standard Trizol (Invitrogen) or QIAzol (QIAGEN), or other analogous protocol for RNA isolation. Alternatively, use RNeasy kit (QIAGEN), or other column-based RNA isolation method. Depending on the starting material, consider the following RNA purification procedures:
  - For small amount of whole blood (less than 100 μl) use 1 ml of Trizol or specific RNA isolation kits (for example, QIAamp RNA Blood Kit, QIAGEN).
  - For large amount of whole blood, preferably perform preliminary PBMC separation using standard procedures (Ficoll density gradient separation) and proceed to C.
  - For large amount of white blood cells, use 1 ml of Trizol (per up to 10^7^ cells). If using column-based RNA isolation method for the large amount of cells, DNase treatment is necessary (according to a manufacturer protocol) since large amounts of genomic DNA significantly affect cDNA synthesis.
  - For small amount of cells (below 100,000 live cells, for example, sorted or bead-separated T or B cells), preferably perform isolation of total RNA shortly after cell acquisition, in order to minimize loss of live cells and mRNA. When using Trizol protocol, add a co-precipitant (e.g., Pellet Paint, Millipore) to the aqueous phase before adding isopropyl alcohol. It is highly desirable that the precipitant forms a single well-defined spot. This provides confidence that some portion of the material will not be washed off by EtOH. Do not discard EtOH used to wash the sample until you are convinced that library preparation has been performed successfully, since some portion of RNA can remain in EtOH.
  All the cell/RNA isolation, cDNA synthesis and first PCR preparation steps should be carried out in a clean DNA/RNAase free room or a PCR box with no contact with any TCR-containing PCR products to prevent contamination. Standard RNA samples handling precautions should be used (gloves, labcoats, filtered tips, and certified RNAase free reagents) to avoid RNA degradation.

Time: 1–2 h.
Pause: RNA can be stored in 70% ethanol at −70°C for at least a year.

### cDNA synthesis and template switch

2. Mix the following in a final volume of 4 μl in a sterile thin-walled reaction tube (mix1).
  **Table:** 

Component	Amount, μl	Final concentration*
RNA	1–3	Maximum 2 μg
cDNA synthesis primer(s) (20 μM)**	0.5–1.5 (0.5 each)	1 μM for each primer
mQ	0–2.5	

*Final concentration/amount in 10 μl after adding mix2 (see Step 5).

**See Table 1 for primers used. Simultaneous synthesis of TCR alpha and beta cDNA is possible (tested for both human and mouse) in case if limited starting material is available. Simultaneous synthesis of IgA, IgM, and IgG heavy chains cDNA is also possible (tested for human).
  Put no more than 1.5–2 μg of total RNA per 10 μl of final reaction volume. For the extra-deep profiling use proportional volume to obtain cDNA from desired amount of starting RNA.
3. Place the reaction tube(s) into a thermal cycler and incubate for 4 min at 70°C and then for 2 min at 42°C to anneal synthesis primer(s).
4. While incubating, mix the following in a separate tube in a final volume of 6 μl (mix2).
  **Table:** 

Component	Amount, μl	Final concentration*
First strand buffer (5×, Evrogen or Clontech)	2	1×
DTT (20 μM)	1	2 μM
5′-template switch adapter (10 μM)	1	1 μM
dNTP solution (10 mM each)	1	1 mM each
Mint reverse transcriptase (10×, Evrogen) or SMARTScribe reverse transcriptase (10×, Clontech)	1	1×

*Final concentration in 10 μl after adding mix 2.
5. Add mix2 to mix1 and mix by pipetting, incubate 40–60 min at 42°C.
  Reverse transcriptases are heat sensitive. Allow the mixture to chill to 42°C after first step denaturation at least for 2 min as described.
  Reverse transcriptases are not equal in their 5′-template switching activity. We have extensive experience with Mint and SMARTScribe reverse transcriptases that provide reliable 5′-template switching.
6. (Optional, for Mint Reverse transcriptase only, to enhance template switching activity) Add 5 μl of IP solution (Evrogen) and incubate at 42°C for additional 1 h.
7. (Optional, see Unique Molecular Identifiers in Appendix) Add 1 μl of Uracyl DNA glycosylase (5 U/μl, New England Biolabs) and incubate 1 h at 37°C.

Time: 2–3 h.
Pause: although cDNA is generally stable, we prefer not to store cDNA longer than several hours at +4°C for the deep profiling experiments. Freezing small amounts of cDNA is undesirable.

### First PCR amplification

8. In a sterile thin-walled tube(s) mix the following in a final volume of 25 μl.
  **Table:** 

Component	Amount, μl	Final concentration
First strand cDNA	1	
Tersus buffer (10×, Evrogen)	2, 5	1×
dNTP (2.5 mM each)	1, 5	0.15 mM each
Primer smart20 (10 μM)	1	0.4 μM
Reverse primer(s) (10 μM)*	1–3 (1 each)	0.4 μM (each)
Tersus polymerase mix (50×, Evrogen)	0.5	1×
mQ	17.5–15.5	

*See Table 1 for primers used. Simultaneous amplification of TCR alpha and beta cDNA is possible (tested for both human and mouse) in case if limited starting material is available. Simultaneous amplification of IgA, IgM, and IgG heavy chains cDNA is also possible (tested for human).
  Put no more than 1 μl of cDNA from the synthesis reaction per 25 μl PCR reaction volume. For the deep profiling, use proportional number of tubes to amplify all the cDNA obtained.
  Polymerase with high fidelity and processivity should be used for amplification.
9. Carry out 18 (when starting from large amount of cells) or 21 (when starting from small amount of cells) cycles of amplification using the following program: 95°C for 20 s, 65°C for 20 s, 72°C for 50 s.
10. Combine all the first step PCR products and purify a portion using the QIAquick PCR purification Kit (or other column-based purification system).

Time: 2–3 h.
Pause: purified first PCR product can be stored at −20°C for a month as a source for the re-amplification of material in the second PCR.

### Second PCR amplification

11. Mix the following in a sterile thin-walled tube in a final volume of 25 μl.
  **Table:** 

Component	Amount, μl	Final concentration
Purified first PCR product	1	
10×polymerase buffer (e.g., Tersus buffer, Evrogen)	2.5	1×
dNTP (2.5 mM each)	1.5	0.15 mM each
Primer Step1 (10 μM)	1	0.4 μM
Reverse primer (10 μM)*	1	0.4 μM
50×polymerase (e.g., Tersus polymerase, Evrogen)	0.5	1×
mQ	17.5	

*See Table 1 for primers used. For primer design options see Sample Barcoding, Unique Molecular Identifiers, and Introducing Diversity at the Ends of the Library in Appendix. In case of simultaneous cDNA synthesis and first PCR amplification of TCR alpha and beta chain libraries, second PCR for TCR alpha and beta chain libraries preparation should be performed in separate reactions. Use an aliquot of purified first PCR product to generate TCR beta library (with beta specific primer) and TCR alpha library (with alpha specific primer).
  Polymerase with high fidelity and processivity should be used for amplification.
12. Carry out amplification using the following program: 95°C for 20 s, 65°C for 20 s, 72°C for 50 s, 9–12 cycles (up to 18–20 cycles if starting from minimal amounts of RNA); final elongation at 72°C for 5 min).
  Purify the PCR products using QIAquick PCR purification Kit (or other column-based purification system) at the same day. This step is important since it removes the residual enzyme activities that can damage the obtained PCR library.

Time: 2 h.
Pause: libraries can be stored at −20°C for weeks before adapter ligation.

### Mixing the barcoded samples for multiplex sequencing

In order to combine several PCR libraries with pre-introduced sample barcodes (see Figure 1 and Sample Barcoding in Appendix for possible options), perform the following:

13. Determine the concentration of each library using the QuBit Fluorometer.
14. Combine samples in a sterile microcentrifuge tube proportionally to the desirable amount of sequencing reads per sample. A total amount of PCR products should be approximately 0.5–1 μg (specify the required amount of the PCR product in a sequencing center).

Alternatively, each sample can be ligated to sequencing adapters with different sample barcodes separately. Samples are mixed in desirable proportions before sequencing.

## Next Generation Sequencing Options

Design of the current protocol is optimized for the Illumina paired end 2 × 150 nt (or 2 × 300 nt for IGs) sequencing as the most reliable way to obtain unbiased TCR/IG repertoire. The paired end sequencing is obligatory when double sample barcodes (see and Sample Barcoding in Appendix) and/or unique molecular identifiers (see Unique Molecular Identifiers in Appendix) are used. If no unique molecular identifiers are used, and sample barcoding is used on the 3′-end of the library only (Figure 1), then single end sequencing is possible. However, only half of obtained sequencing reads will contain the CDR3 region.

Protocol also suits well the Roche 454 sequencing technology. Frequent length-errors in reading homogenous oligonucleotide stretches on this platform should be kept in mind, and proper error-correction algorithms utilized (10).

In order to use Illumina paired end 2 × 100 nt sequencing for TCRs, the only required modification is that multiplexed J-segment-specific primers should be used instead of the reverse primer in the second PCR amplification step. This minor multiplexing within limited number of PCR cycles does not lead to essential quantitative bias and allows sequence to start closer to the CDR3 region of interest, as described (10, 16). For IG’s heavy chain, the universal J-segment-specific primer (Table 1) is close to CDR3 already and no modifications are necessary.

**Table 1:** **Oligonucleotides**.

Primer	Application	Sequence*,**
**FIRST STRAND cDNA SYNTHESIS**		
Switch_oligo	5′adapter: template switch adapter, universal for all libraries	AAGCAGTGGTATCAACGCAGAGTAC(XXXXX)TCTT(rG)_5_
SmartNNN	Alternative template switch adapter with unique molecular identifier (see Unique Molecular Identifiers in Appendix), universal for all libraries	AAGCAGUGGTAUCAACGCAGAGUNNNNUNNNNUNNNNUCTT(rG)_5_
AC1R	Primer for cDNA synthesis, human TCR alpha mRNA	ACACATCAGAATCCTTACTTTG
BC1R	Primer for cDNA synthesis, human TCR beta mRNA	CAGTATCTGGAGTCATTGA
Mus_alfa_synt1	Primer for cDNA synthesis, mouse TCR alpha mRNA	TTTCGGCACATTGATTTG
BC_mus_syn1	Primer for cDNA synthesis, mouse TCR beta mRNA	CAATCTCTGCTTTTGATG
HCA-rt	Primer for cDNA synthesis, human IgA heavy chain mRNA	GTCCGCTTTCGCTCCAGG
HCM-rt	Primer for cDNA synthesis, human IgM heavy chain mRNA	GATGTCAGAGTTGTTCTTG
HCG-rt	Primer for cDNA synthesis, human IgG heavy chain mRNA	GTGTTGCTGGGCTTGTG
**FIRST PCR AMPLIFICATION**		
Smart20	Step-out primer 1. Anneals on the switch_oligo, universal for all libraries	CACTCTATCCGACAAGCAGTGGTATCAACGCAG
AC2R	Nested primer 1, human TCR alpha library	TACACGGCAGGGTCAGGGT
BC2R	Nested primer 1, human TCR beta library	TGCTTCTGATGGCTCAAACAC
Mus AV2 rev	Nested primer 1, mouse TCR alpha library	GGTGCTGTCCTGAGACCGAG
BC4_mus_Rev	Nested primer 1, mouse TCR beta library	GATGGCTCAAACAAGGAGACC
HCA-n1	Nested primer 1, human IgA heavy chain library	GCGATGACCACGTTCCCATCT
HCM-n1	Nested primer 1, human IgM heavy chain library	GTGATGGAGTCGGGAAGGAAG
HCG-n1	Nested primer 1, human IgG heavy chain library	GAAGTAGTCCTTGACCAGGCA
**SECOND PCR AMPLIFICATION**		
Step_1	Step-out primer 2, from the Smart20, universal for all libraries	(N)_2–4_(XXXXX)CACTCTATCCGACAAGCAGT
Hum bcj	Nested primer 2, human TCR beta	(N)_2–4_(XXXXX)ACACSTTKTTCAGGTCCTC
Hum acj	Nested primer 2, human TCR alpha	(N)_2–4_(XXXXX)GGGTCAGGGTTCTGGATAT
Mus bcj	Nested primer 2, mouse TCR beta	(N)_2–4_(XXXXX)GGAGTCACATTTCTCAGATCCT
Mus acj	Nested primer 2, mouse TCR alpha	(N)_2–4_(XXXXX)CAGGTTCTGGGTTCTGGATGT
IGHJ-r1	Nested primer 2, human IG heavy chain (universal for IgA, IgG, and IgM)	(N)_2–4_(XXXXX)GAGGAGACGGTGACCRKGGT

*XXXXX: optional sample barcode (see Figure 1, and Sample Barcoding in Appendix for details and Supplementary Material for barcodes). *U* = d*U* (deoxyuridine).

**(N)_2–4_ – optional. Random nucleotides (“N”) are introduced at the 5′ end of final library in order to generate diversity for better cluster identification on Illumina sequencer (see Introducing Diversity at the Ends of the Library in Appendix for details).

Alternative strategy is that sequences for Illumina flow cell and custom sequencing primers can be introduced in the course of amplification (not shown on Figure 1). Although potentially beneficial, it requires thorough design in cooperation with sequencing centers.

This protocol is not adopted for Ion Torrent as these sequencing machines have limitations in the maximal length of analyzed sequencing library. Multiplex PCR mix for the V-segment is required for Ion Torrent library preparation, albeit leads to significant quantitative bias during amplification (10).

To provide better cluster differentiation, ask sequencing facility to spike the library with 10–30% of PhiX and/or design primers as described in Introducing Diversity at the Ends of the Library in Appendix.

Size selection on agarose gel after ligation of adapters is strongly recommended since even minor amounts of short non-specific PCR products can significantly reduce target sequences output.

## Software Analysis of NGS Data

Output NGS data on TCR/IG profiling contain numerous errors accumulated during reverse transcription, PCR amplification, and sequencing. For the latter, higher Phred quality score only means lower frequency of sequencing errors. Thus, high sequence quality does not guarantee absence of sequencing errors. Generally, the more we sequence, the more erroneous TCR/IG variants we generate. Without appropriate error-correction, NGS data can generate artificial TCR/IG diversity exceeding the native diversity of complex input library up to several-fold (10).

Several approaches were proposed to correct the PCR and high quality sequencing errors in TCR datasets, suggesting to filter off low frequency TCR variants (8), to filter off the low abundance variants with single mismatch comparing to the major clonotypes (7), or to correct single mismatch errors in germline segments by mapping to the major clonotypes (10). Low quality sequences can be either filtered off (7, 8) or mapped to the high quality ones in order to rescue quantitative information (10).

**Figure 2:**
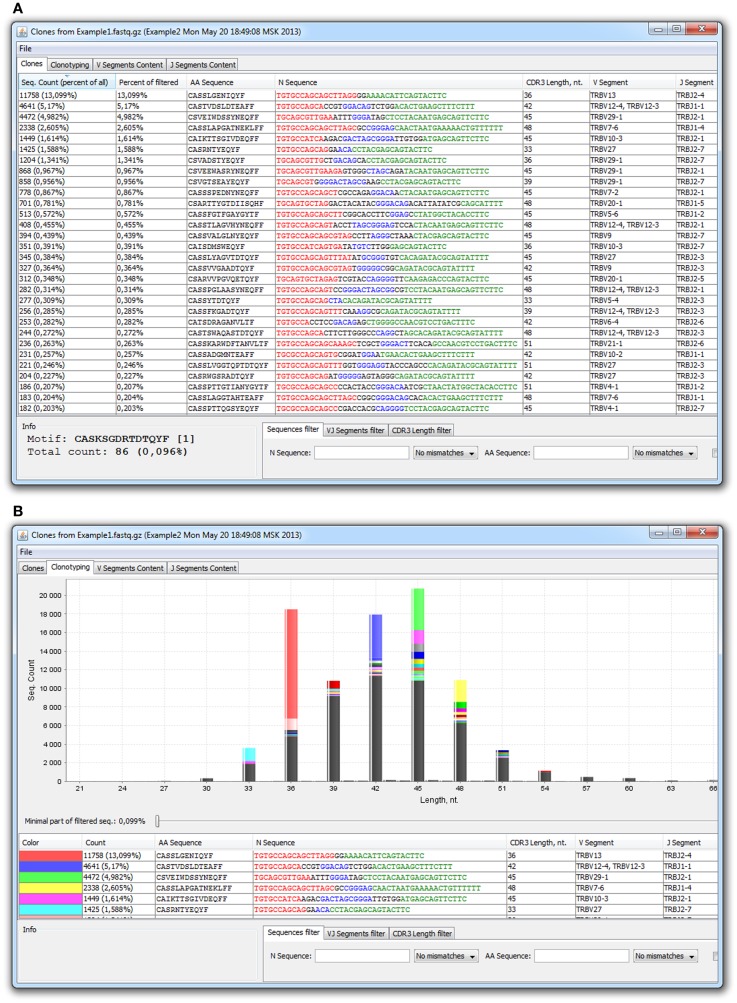
**MiTCR-viewer outputs for the analyzed TCR beta dataset**. **(A)** Table with clonotypes. **(B)** *In silico* spectratyping.

There are currently three available software packages for NGS TCR data analysis: IMGT/HighV-QUEST web service^2^, Decombinator (28), and our new software, named MiTCR^3^ (29). Note that IMGT/HighV-QUEST is limited to only 50,000–150,000 sequences per batch and thus it is hardly suitable for the analysis of deep NGS profiling data. MiTCR is the only software package that considers sequence quality, performs correction of PCR and sequencing errors, and rescues low quality sequencing data. Two basic error-correction modes are currently implemented, aiming either to eliminate maximal number of accumulated errors, or to preserve maximal original TCR diversity, albeit with less efficient error-correction. Moreover, analysis parameters can be tuned by user in a wide range to obtain optimal result for the particular experimental task. Output format is a tab-delimited file or a special *.cls file for the MiTCR-Viewer software (Figure 2).

## Expected Results

### RNA

The quality and quantity of obtained RNA is critical for the library generation. Quality of total RNA is evaluated by two visible bands on electrophoresis (or two highest peaks on Agilent Bioanalyzer) corresponding to 18S and 28S rRNA. The relative amount of two bands should be between 1:2 and 1:1. The expected yield is 1–3 μg of total RNA from one million of PBMC when using Trizol protocol. If starting material is limited (10,000 cells or less) RNA should be completely used in one cDNA synthesis reaction without analyzing by electrophoresis.

### Number of PCR cycles

In order to preserve natural TCR/IG diversity of the sample it is important to minimize the number of PCR cycles used for library preparation. In our system, maximal number of PCR cycles should be 18 for the first and 12 for the second amplification step if starting from 2 μg of total RNA. A well visible band is observed on electrophoresis after 12 cycles of second PCR amplification (that is at least 50 ng of PCR product per 25 μl reaction). For a minimum amount of starting material (below 10,000 cells) the maximum number of PCR cycles should be 21 for the first and 18–20 for the second amplification step. If the number of cycles needed to obtain a visible band is higher, this may indicate that low number of molecules has successfully entered amplification, thus leading to uncertain detection of CDR3 clonotypes of the input sample.

### Sequencing output and analysis

With the use of the proposed protocol, at least three million of high quality CDR3-containing sequencing reads from a paired end MiSeq run and at least 100 million CDR3-containing sequencing reads from one lane of paired end HiSeq 2,000/2,500 run are expected. The number of different clonotypes depends on the nature and amount of starting material. For example, profiling of 5–10 million human PBMC cells using 1/10 of HiSeq 2000 Illumina lane (at least 10 million CDR3-containg reads) can yield from 0.5 to 2.5 million TCR beta CDR3 clonotypes after appropriate error-correction.

## Conflict of Interest Statement

The authors declare that the research was conducted in the absence of any commercial or financial relationships that could be construed as a potential conflict of interest.

## Supplementary Material

The Supplementary Material for this article can be found online at http://www.frontiersin.org/Journal/10.3389/fimmu.2013.00456/abstract

## Footnotes

^1^http://www.imgt.org/

^2^http://www.imgt.org/IMGTindex/IMGTHighV-QUEST.html

^3^http://mitcr.milaboratory.com/

## Appendix

### Sample barcoding

When sequencing multiple samples, it is recommended to introduce sample barcodes during the library preparation process. This allows to minimize cross-sample contamination and to treat all samples as the single one when ligating Illumina adapters. It is possible to introduce sample barcodes on different stages (See Figure 1). One of the best ways is to use 5′-template switch adapters with built-in sample barcodes, thus labeling each sample at the very first library preparation step. Alternatively/additionally, 5′-end sample barcode can be introduced at the 5′-end of the *Step-out primer 2* (see Table 1). We also recommend introduction of sample barcodes within the reverse primers used in the second amplification step (*hum bcj*, *hum acj*, *mus bcj*, *mus acj*, or *IGHJ-r1*, see Table 1). Using this approach, each sample is barcoded at both ends of the library. This is crucial when accurate comparison of two or more samples is required, as we observe different levels of swapping ends between molecules in course of standard Illumina library preparation stage and presumably on the solid phase of the sequencer, during bridge amplification. For your convenience, we have generated a list of 5-nucleotide sample barcodes, which differ from each other by at least two nucleotides (see Supplementary Material), thus minimizing the chance of barcode misinterpretation if the single error occurs during sample preparation or sequencing.

### Unique molecular identifiers

Unique molecular identifiers can be introduced as random oligonucleotides at the very first amplification (or cDNA synthesis) step of library preparation (30). Each molecule that successfully enters amplification becomes labeled by a unique combination of nucleotides – a molecular identifier. Thus each TCR/IG CDR3 sequence variant in the output NGS dataset is characterized by a number of distinct molecular identifiers indicating the number of such cDNA molecules that have entered the PCR amplification. This approach allows to correct the PCR bias that occurs during amplification and to count mRNA/cDNA molecules of each type directly, which makes the TCR/IG repertoire analysis even more quantitative. Unique molecular identifiers consisting of 12 random nucleotides (which give approximately 17 million unique variants) can be introduced within the 5′-template switch adapter (Table 1, SmartNNN). This template switch adapter also contains multiple deoxyuridine nucleotides. After cDNA synthesis, Uracyl DNA glycosylase treatment allows to eliminate SmartNNN, thus preventing possible exchange of unique molecular identifiers during following PCR amplification (30).

### Introducing diversity at the ends of the library

The common problem with sequencing PCR products by Illumina is the presence of the same nucleotides in the beginning of most sequencing reads. This can lead to a fail of a sequencing run as Illumina software cannot discriminate adjacent clusters, which produce identical fluorescent signals during the first several sequencing cycles. The common solution used by sequencing centers is spiking the sequencing library by PhiX library containing random DNA fragments. However, in this case, the number of obtained target sequences is decreased by at least 30%. To avoid this problem we introduce two to four random nucleotides (“N”) to the 5′ end of the primers used in the second amplification step (see Table 1). Preferably, the number of “N” nucleotides flanking the library should be different for the samples mixed on the same Illumina lane, in order to generate additional diversity of starting sequencing steps and to avoid identical nucleotides being present in the same positions, which may alter Illumina sequencing quality. If one sample is sequenced per Illumina lane and no sample barcodes are used, it is recommended to use a mixture of three identical primers, each containing a different number of “N” nucleotides at the 5′ end – e.g., (N)_2_ Step1/(N)_3_ Step 1/(N)_4_ Step1, the same with the reverse primer (see Table 1).
