# Supplementary material for: Preparing Unbiased T-Cell Receptor and Antibody cDNA Libraries for the Deep Next Generation Sequencing Profiling
Source: Front Immunol. 2013 Dec 23;4:456. doi: 10.3389/fimmu.2013.00456 (PMC3870325; doi:10.3389/fimmu.2013.00456)
Supplement: Supplementary file 1 [file 51408_Chudakov_DataSheet1.DOC]

**Supplementary Table 1.**

160 5-digit sample barcodes.

Barcodes differing by less than 2 nucleotides are excluded.

For each sample, the same or 3 different barcodes can be introduced within the 5’ switch adapter, and within 5’ and 3’ primers used for the 2^nd^ PCR amplification step.

Primer names can include the sample barcode index, for example:

Smart_i101

Step_ i176

TRBC_H_i232

| AAACT | i101 |
| --- | --- |
| AAAGA | i102 |
| AACGG | i103 |
| AACTC | i104 |
| AAGAG | i105 |
| AAGCC | i106 |
| AAGGT | i107 |
| AATGC | i108 |
| AATTG | i109 |
| ACAAT | i110 |
| ACCAA | i111 |
| ACCCT | i112 |
| ACCTG | i113 |
| ACGCG | i114 |
| ACGGC | i115 |
| ACTCC | i116 |
| ACTGA | i117 |
| AGACA | i118 |
| AGAGT | i119 |
| AGATG | i120 |
| AGCAC | i121 |
| AGCCG | i122 |
| AGCTA | i123 |
| AGGCT | i124 |
| AGGGA | i125 |
| AGGTC | i126 |
| AGTAA | i127 |
| AGTGG | i128 |
| AGTTT | i129 |
| ATACC | i130 |
| ATAGG | i131 |
| ATATT | i132 |
| ATCGC | i133 |
| ATGAC | i134 |
| ATGCA | i135 |
| ATGTG | i136 |
| ATTCG | i137 |
| ATTGT | i138 |
| ATTTC | i139 |
| CAAGG | i140 |
| CACAG | i141 |
| CACCA | i142 |
| CAGAT | i143 |
| CAGCG | i144 |
| CATAA | i145 |
| CATCT | i146 |
| CATTC | i147 |
| CCATG | i148 |
| CCCAT | i149 |
| CCCGC | i150 |
| CCGAA | i151 |
| CCGCT | i152 |
| CCGGG | i153 |
| CCGTC | i154 |
| CCTAG | i155 |
| CCTGT | i156 |
| CGAAG | i157 |
| CGACT | i158 |
| CGAGC | i159 |
| CGCAA | i160 |
| CGCGG | i161 |
| CGCTT | i162 |
| CGGCC | i163 |
| CGGGT | i164 |
| CGTAT | i165 |
| CGTCG | i166 |
| CGTGA | i167 |
| CTAAA | i168 |
| CTACG | i169 |
| CTAGT | i170 |
| CTATC | i171 |
| CTCCT | i172 |
| CTCTG | i173 |
| CTGAG | i174 |
| CTGGA | i175 |
| CTGTT | i176 |
| CTTAC | i177 |
| CTTGG | i178 |
| CTTTA | i179 |
| GAAAT | i180 |
| GAATC | i181 |
| GACAA | i182 |
| GACCC | i183 |
| GACTT | i184 |
| GAGCA | i185 |
| GAGGG | i186 |
| GATAC | i187 |
| GATCG | i188 |
| GATGT | i189 |
| GCAAA | i190 |
| GCAGT | i191 |
| GCCCA | i192 |
| GCCGG | i193 |
| GCCTC | i194 |
| GCGAG | i195 |
| GCGCC | i196 |
| GCGGA | i197 |
| GCGTT | i198 |
| GCTCT | i199 |
| GCTGC | i200 |
| GCTTA | i201 |
| GGACC | i202 |
| GGAGG | i203 |
| GGATA | i204 |
| GGCAT | i205 |
| GGCGC | i206 |
| GGCTG | i207 |
| GGGAC | i208 |
| GGGCG | i209 |
| GGTAG | i210 |
| GGTCA | i211 |
| GGTTC | i212 |
| GTAAG | i213 |
| GTAGC | i214 |
| GTCAC | i215 |
| GTCCG | i216 |
| GTCGT | i217 |
| GTCTA | i218 |
| GTGAA | i219 |
| GTGCT | i220 |
| GTGTC | i221 |
| GTTAT | i222 |
| GTTCC | i223 |
| GTTGA | i224 |
| GTTTG | i225 |
| TAAAG | i226 |
| TAACC | i227 |
| TAATA | i228 |
| TACAC | i229 |
| TACGT | i230 |
| TACTG | i231 |
| TAGAA | i232 |
| ~~TAGGC~~ | ~~i233~~ |
| TAGTT | i234 |
| TATAT | i235 |
| TATGA | i236 |
| TCATC | i237 |
| TCCAG | i238 |
| TCCTA | i239 |
| TCGAC | i240 |
| TCGTG | i241 |
| TCTGG | i242 |
| TCTTT | i243 |
| TGAAA | i244 |
| TGATT | i245 |
| TGCCT | i246 |
| TGCTC | i247 |
| TGGCA | i248 |
| TGTCC | i249 |
| TGTGT | i250 |
| TGTTA | i251 |
| TTAAT | i252 |
| TTATG | i253 |
| TTCCC | i254 |
| TTCGA | i255 |
| TTCTT | i256 |
| TTGCG | i257 |
| TTTAG | i258 |
| TTTCT | i259 |
| TTTGC | i260 |
